# The role of radiological interventions in hepatocellular carcinoma before liver transplantation: a surgical perspective

**DOI:** 10.3389/fsurg.2025.1594579

**Published:** 2025-10-08

**Authors:** Altan Alim, Derek DuBay

**Affiliations:** School of Medicine, Section of Organ Transplantation and Hepatobiliary Surgery, Greenville Prisma Hospital, University of South Carolina, Greenville, SC, United States

**Keywords:** liver transplantation, TACE (transarterial chemo-embolization), TARE (transarterial radio-embolization), hepatocecllular carcimoa (HCC), living donor liver transplantation, downstaging protocol, bridging protocol

## Abstract

Liver transplantation (LT) remains the gold standard treatment for patients with unresectable hepatocellular carcinoma (HCC) within or, in select cases, beyond the Milan criteria. However, with the increasing complexity of HCC management and the scarcity of donor organs, the role of liver directed therapies have gained prominence in optimizing patient outcomes. Downstaging therapies and bridging therapies have become essential components of HCC management. This review explores the pivotal role of interventional radiology interventions, including thermal ablation techniques (radiofrequency ablation, microwave ablation) and transarterial therapies (transarterial chemoembolization, transarterial radioembolization), in the pre-transplantation setting. These therapies not only improve LT eligibility for patients exceeding traditional tumor criteria but also enhance survival by maintaining disease control and reducing dropout rates from the LT waiting list. The review further discusses the complexities of patient selection, contraindications, and the evolving strategies in locoregional therapy to maximize LT outcomes. Liver directed therapies, through both downstaging and bridging, are integral to managing HCC, offering significant benefits in post-transplant survival, while ensuring that LT is conducted based on appropriate indications.

## Introduction

Liver transplantation (LT) remains the gold standard treatment for patients with hepatocellular carcinoma (HCC) within the Milan criteria or, in selected cases, beyond the Milan criteria without evidence of metastasis (1). Living donor liver transplantation (LDLT) is particularly beneficial for patients exceeding the Milan criteria who are unable to access deceased donor liver grafts through national organ allocation systems, providing satisfactory survival outcomes (1, 2).

For patients without access to a living donor (or in countries where LDLT is not the predominant practice, such as the United States) and for those with HCC exceeding the Milan criteria, innovative strategies have emerged to ensure eligibility for LT. These include downstaging therapies aimed at reducing tumor burden to meet LT criteria, as well as bridging therapies designed to prevent tumor progression or metastasis during prolonged waiting times on the LT list. These approaches have recently gained significant attention (3). It is strongly recommended that all patients with HCC on the liver LT waiting list undergo comprehensive evaluation to determine their eligibility for bridging or downstaging therapies. These interventions play a critical role in minimizing the risk of tumor progression, maintaining LT candidacy, and improving overall outcomes (4).

Moreover, liver directed therapies, such as downstaging or bridging, are increasingly employed as primary treatment modalities in cases where LT or surgical resection is not feasible (3). Among these, thermal ablation techniques and transarterial interventions have emerged as the most widely utilized and effective options.

### Definition and terminology

Bridging and downstaging therapies play a crucial role in maintaining LT eligibility during long waiting times.

Bridging therapy: This term refers to the therapeutic management of LT-eligible patients who meet the Milan criteria while they remain on the waiting list. During this period, patients with HCC face a significant risk of being removed from the list due to tumor progression. For this reason, bridging treatments are advised, particularly for those anticipated to wait for a LT for more than six months. Notably, around 22% of HCC patients are delisted from the LT waiting list, with tumor progression accounting for approximately half of these cases (3, 5). Transarterial interventions are utilized in approximately 70% of patients on the LT waiting list due to HCC in the United States. These procedures play a crucial role in preventing HCC tumor progression, thereby ensuring that patients remain eligible for LT (6).

Downstaging: The concept of “downstaging” refers to therapeutic strategies targeting HCC lesions in patients whose tumor burden exceeds established LT criteria. The primary goal is to reduce tumor size and burden, enabling these patients to meet the criteria and achieve post-transplant survival rates comparable to those who were initially within the accepted criteria and did not require downstaging (7). As outlined in the European Association for the Study of Liver (EASL) guidelines, LT is considered a viable option for patients whose HCC initially exceed the Milan criteria only if their tumors can be successfully downstaged to meet the Milan criteria (3, 8).

### Treatment modalities

Historically, locoregional therapies trace their roots back to the use of ethanol injection, a technique employed in unresectable tumors during an era when LT and advanced interventional radiological (IR) procedures were not yet widespread (9). Ethanol, a chemical with high cytotoxicity against all cell types, was administered over several sessions, aiming to reduce tumor burden.

With technological advancements in the mid-1990s, radiofrequency ablation (RFA) and microwave ablation (MWA) emerged as prominent thermal ablation methods (10, 11). Simultaneously, transarterial approaches such as transarterial chemoembolization (TACE) and transarterial radioembolization (TARE) gained popularity (12, 13).

1. *Thermal Ablations*

### Radiofrequency ablation (RFA)

First introduced in the early 1990s, RFA remains the most well-established and widely utilized thermal ablation technique for HCC. In patients on the LT waiting list, RFA plays a crucial role as a bridging therapy, effectively mitigating the risk of tumor progression that could otherwise lead to delisting. Additionally, by controlling local tumor burden, RFA helps prevent distant metastasis, thereby preserving the patient's eligibility for LT and improving overall prognosis (14).

Using a probe to deliver targeted heat, RFA is more effective for achieving tumor necrosis in small lesions compared to larger tumors. For tumors measuring ≤3 cm, RFA achieves necrosis rates as high as 76%, with even better outcomes in smaller lesions (3). However, to optimize necrosis rates, repeated sessions are often recommended.

One limitation of RFA is its propensity to produce high heat, which can be problematic in tumors located near major vascular structures, bile ducts, or subcapsular regions adjacent to other organs. The potential for complications in such cases remains a concern, limiting its applicability in anatomically challenging scenarios.

### Microwave ablation (MWA)

**Figure 1:**
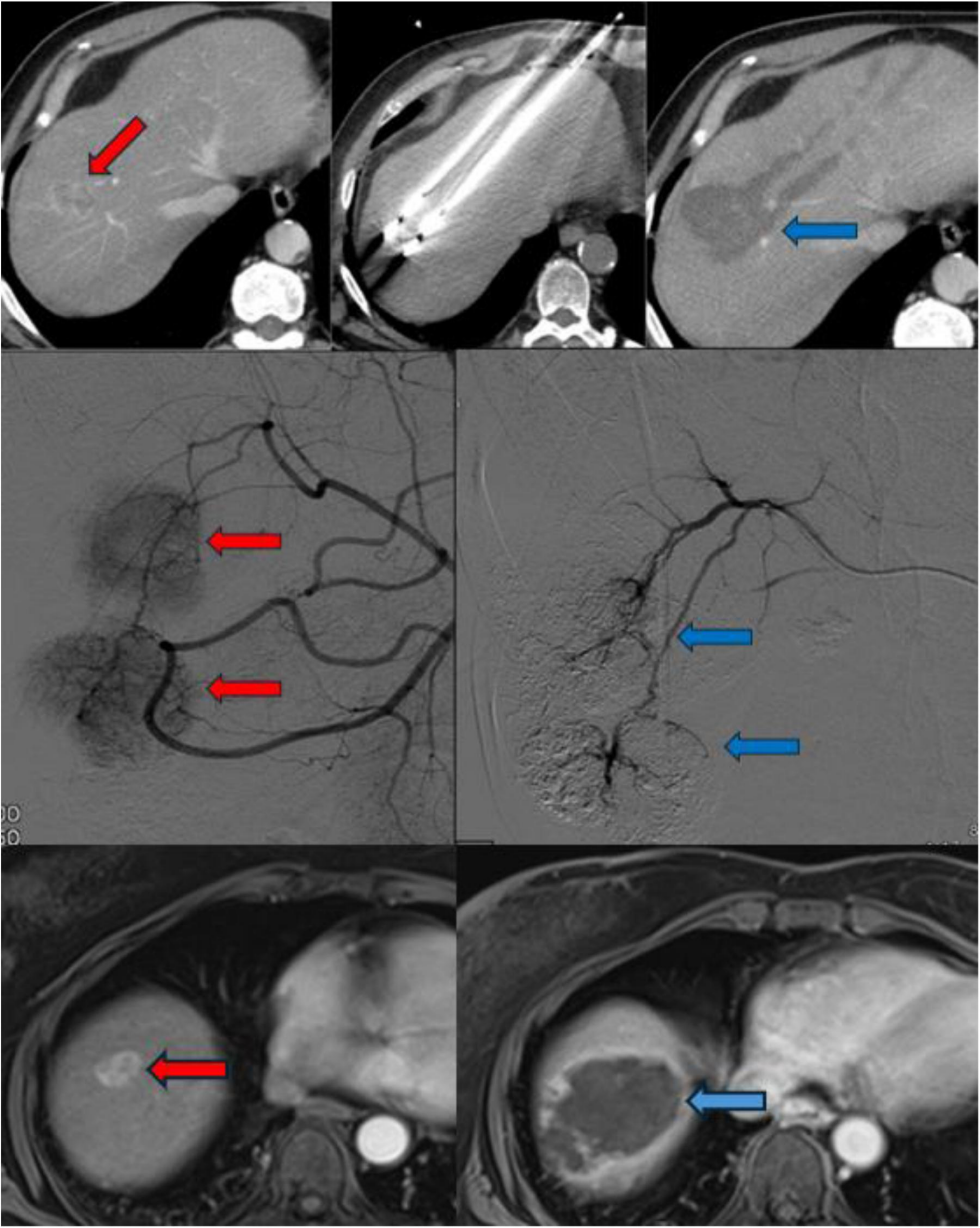
The radiological imaging of patients who underwent MWA (upper column), TACE (middle column) and TARE (lower column). The red arrows indicate to HCC foci and the blue arrows indicate after procedures appearance.

Technically similar to RFA, MWA has gained preference in cases where tumors are located near critical anatomical structures due to its ability to generate less heat diffusion (15). MWA rapidly heats tissues by inducing movement in water molecules, allowing for faster and higher temperatures (Figure 1). Unlike RFA, MWA causes less surrounding tissue damage and reduced inflammatory reactions, making it a safer option in sensitive locations.

MWA is considered more effective for larger tumors due to the ability to utilize more than 1 probe and bracket the tumor. However, its ability to achieve complete necrosis in lesions >4 cm remains debatable. Post-transplant pathological studies have shown a complete necrosis rate of approximately 78% in patients who underwent single-session MWA prior to LT. Therefore, similar to RFA, repeated sessions are recommended to improve efficacy (3).

1. Intra-arterial embolization interventions

### Transarterial chemoembolization (TACE)

TACE is a widely utilized liver directed therapy for HCC, particularly in patients awaiting LT. This procedure involves the intra-arterial administration of drug-eluting beads (DEBs) under angiographic guidance. The catheter is placed as selectively as possible to isolate the tumor's blood supply, thereby maximizing localized chemotherapy delivery.

Doxorubicin is the most commonly used chemotherapeutic agent, exerting its cytotoxic effects by intercalating DNA and inhibiting cell division. Additionally, cisplatin, mitomycin C, and irinotecan are frequently employed in TACE protocols. The choice of chemotherapy regimen is determined based on tumor burden, patient performance status, and prior treatment response.

Depending on the vascular anatomy, selective hepatic embolization targets the right or left hepatic arteries separately, while superselective embolization aims at smaller branches directly supplying the tumor (Figure 1). The choice of DEB size varies across studies, with smaller particles (100–300 µm or 300–500 µm) typically used first, followed by larger ones (500–700 µm) (16).

### Patient selection and contraindications

While TACE serves as an effective bridging therapy, patient selection remains critical, particularly in the pre-transplant setting, as embolization can precipitate hepatic decompensation in cirrhotic patients. Absolute contraindications to TACE include:

- Decompensated cirrhosis as evidenced by a Child-Pugh score ≥ 8, medically refractory ascites or bilirubin >3.0
- Extensive tumor burden with involvement of both liver lobes
- Vascular abnormalities, such as arteriovenous fistulas impairing intra-arterial treatment feasibility
- Severely impaired portal vein flow
- Renal dysfunction (serum creatinine > 2 mg/dl or creatinine clearance < 30 ml/min) (17)
- Relative contraindication is centrally positioned larger tumors with multiple arterial feeders

TACE should not be repeated if substantial tumor necrosis is not achieved after two sessions or if there is evidence of disease progression, liver function deterioration, or worsening performance status (3, 18).

### Post-TACE effects and risks

A well-documented effect of TACE is the induction of ischemia, which stimulates vascular endothelial growth factor (VEGF) release, potentially promoting tumor growth through neo-angiogenesis (18). Additionally, TACE may cause endothelial injury to the hepatic artery, increasing the risk of hepatic artery thrombosis (HAT) (19, 20, 21). A systematic review of 1,122 patients across 14 studies identified a significant association between pre-LT TACE and post-transplant hepatic artery complications (OR: 1.57; 95% CI: 1.09–2.26; *p* = 0.02) (15).

However, some studies have reported no significant increase in hepatic artery complications following TACE in LT recipients (22, 23). Despite these conflicting findings, the impact of pre-transplant TACE should be carefully evaluated on a case-by-case basis.

### Transarterial radioembolization (TARE)

TARE, also referred to as radioembolization, y^90^ or selective internal radiation therapy (SIRT), involves the selective intra-arterial administration of microspheres loaded with radioactive compounds—most commonly Yttrium-90 (^90Y) or Lipiodol labeled with Iodine-131 (^131I) or Rhenium-188 (^188Re)—via percutaneous access. Among these, Yttrium-90 has become the most widely used radionuclide. It is a pure *β*-emitter, characterized by a short half-life (64.2 h) and limited tissue penetration (average 2.5 mm, maximum 11 mm) (24). Deployment devices aid in the administration of Y^90^ directly into the tumor minimizing contamination to non-targeted areas (Figure 1).

The size of the microspheres varies depending on the radioactive compound used, typically ranging between 20 and 60 µm. Compared to TACE microspheres, TARE microspheres are significantly smaller, allowing them to pass through smaller arterioles, thereby reaching deeper into the tumor tissue and exerting their therapeutic effect through localized radiation. Unlike other embolization-based treatments such as TACE, radioembolization does not induce a macroembolic effect. Consequently, both its therapeutic benefits and potential toxicities are directly related to the radiation dose delivered by the microspheres, rather than to any ischemic effect caused by arterial occlusion (25).

### Patient selection and contraindications

Standardized eligibility criteria for TARE have been established based on manufacturer guidelines and retrospective clinical studies. Patients considered suitable for TARE generally meet the following conditions (26).

- Performance status: Eastern Cooperative Oncology Group (ECOG) 0–2
- Liver function: Aspartate aminotransferase (AST)/alanine aminotransferase (ALT) < 5× upper limit of normal, total bilirubin <2 mg/dl
- Renal function: Normal creatinine levels, as contrast agents are required for hepatic arteriography and catheterization
- HCC-specific criteria: Child-Turcotte-Pugh (CTP) class A or B7
- Tumor burden: Non-infiltrative tumor type, tumor volume <70% of the targeted liver volume, or tumor nodules that are not too numerous to count
- Relative contraindication are central positioned tumors adjacent to major lobar bile ducts

### Post-TARE effects and risks

The most frequently reported side effect of TARE is post-radioembolization syndrome (PRS), which includes fatigue, nausea, vomiting, abdominal pain, appetite loss, and weight loss. Its incidence ranges from 20% to 70%, peaking within the first two weeks following treatment. A less common but clinically significant adverse event is radioembolization-induced liver disease (REILD). A comprehensive review evaluating 19 studies reported REILD incidence ranging from 0% to 11% in HCC patients and 0% to 20% in patients with metastatic liver disease. It is ideal for an HCC patient to be actively listed for LT when there are concerns about REILD. Other uncommon complications of TARE include gastroduodenal ulceration, biliary toxicity, and radiation pneumonitis (26).

### Considerations for sequential use of TACE and TARE

A critical consideration when planning sequential locoregional therapies is the order of administration. If a patient has previously undergone TACE, the subsequent use of TARE is not recommended. This is due to the larger sphere size of TACE embolic agents, which induce substantial arterial occlusion, potentially hindering the delivery of smaller TARE microspheres to the target tissue.

Conversely, if TARE is administered first, its smaller particle size and gradual volume reduction due to radioactive decay minimize its embolic effect, allowing for subsequent TACE without obstruction. Therefore, TACE can be safely performed after TARE, as its embolization process does not interfere with prior radioembolization (3).

### Considerations in preparing an HCC patient for LT

When preparing a patient with HCC for LT, it is essential to gather relevant clinical data systematically. The integration of these data into a unified risk assessment model is crucial for determining the most appropriate treatment strategy.

1. Donor Availability and Timing of LT

The primary consideration in LT planning is the availability of a suitable graft. In countries where LDLT predominates, such as those in Asia, over 90% of transplants are performed using living donors. This offers significant advantages, including precise surgical timing, optimal preoperative stabilization, and the ability to act swiftly (1, 2). However, the risk of major complications and donor mortality remains a critical limiting factor. As a general principle, recipient selection criteria often require a projected five-year survival rate of at least 50% to justify the risks associated with donor morbidity (2, 27).

The expected wait time for a deceased donor organ is a key factor in treatment decisions for HCC patients with end-stage liver disease (ESLD). If an organ is likely to become available soon—either from a waiting list or a living donor—LT may be prioritized over liver directed therapies to HCC. Conversely, if the estimated waiting time exceeds three months, liver directed therapy should be considered to control local progression and prevent distant metastasis. Additionally, in cases where high tumor burden precludes listing, downstaging procedures should be strongly considered and may be required to attain priority for deceased donor liver allocation.

1. Severity of Underlying Liver Disease (MELD and Child-Pugh Classification)

The severity of ESLD is one of the most critical factors in determining the suitability of downstaging or bridging therapy. In decompensated cirrhotic patients with deteriorating liver function, transarterial interventions pose a significant risk of further decompensation due to both chemotherapy-related toxicity and procedure-induced parenchymal loss (28). These interventions may ultimately lead to mortality before LT can be performed. Current guidelines suggest that in patients with high MELD or Child-Pugh scores, thermal ablation techniques should be prioritized whenever indicated (e.g., Downstaging requirements) and feasible.

1. Tumor Aggressiveness, Size, and Risk of Metastasis

The most commonly used criteria for assessing HCC lesions include:

a. Tumor growth characteristics
b. Tumor burden
c. Presence of macrovascular/microvascular invasion
d. Alpha-fetoprotein (AFP) levels and *Δ* AFP
e. Pathologic differentiation of the tumor

Many transplant centers have developed their own institutional algorithms for the management and preparation of LT candidates with HCC (2). However, the most fundamental principle remains the necessity of individualized, case-based assessment for each HCC patient. In alignment with this principle, our center has established a standardized clinical guideline for the initial evaluation of HCC patients, which serves as a framework while allowing for individualized decision-making (Table 1).

**Table 1:** The first approach for cirrhotic patients with HCC.

Criteria	AFP level & other factors	Initial approach	Follow-up & outcome
In Milan Criteria	AFP < 300	Waiting List	LT if organ available IR (Bridging) if waiting time >3 months
	AFP > 300	IR (Downstaging)	After 3 months: If AFP ↓ & No metastasis → LT If AFP ↑ → Continue IR & Follow-up
Beyond Milan& In UCSF	AFP < 300	Waiting List	LT if organ available IR (Bridging) if waiting time > 6 months
	AFP > 300	IR (Downstaging)	After 6 months: If AFP ↓ & No metastasis → LT If AFP ↑ → Continue IR & Follow-up
Beyond UCSF	AFP high or low	IR (Downstaging) Biopsy	If Poorly Differentiated → Non-LT options If Well-Moderate differentiated After 6 months: If AFP ↓ & No metastasis → LT If AFP ↑ → Continue IR & Follow-up
Exceptions PVTT Infiltrative HCC Massive HCC	AFP high or low	IR (Downstaging) Biopsy	If Poorly Differentiated → Non-LT options If Well-Moderate differentiated After 6 months: AFP ↓ & No metastasis → LT If AFP ↑ → Continue IR & Follow-up
Systemic Disease HVTT Diffuse HCC Distant metastasis	AFP high or low	IR (Downstaging)	Non-LT options, Repeated IR & Follow-up

LT remains the preferred treatment for HCC patients; however, certain tumor characteristics limit its applicability. Macrovascular invasion and elevated alpha-fetoprotein (AFP) levels are among the most critical factors restricting LT eligibility (1). Additionally, tumor growth patterns and elevated AFP levels serve as important prognostic indicators, particularly in assessing the risk of microvascular invasion. Infiltrative or massive HCC lesions carry a high risk of metastasis, making primary disease control via liver directed therapies a more rational approach than immediate LT (1, 2). For patients with HCC within the UCSF criteria, our approach parallels that used for Milan criteria-compliant tumors, provided that radiological imaging demonstrates a well-defined, non-infiltrative tumor pattern without macrovascular invasion. In cases where AFP levels exceed 300 ng/ml, irrespective of tumor size, we advocate for an initial liver directed therapy, followed by re-evaluation with imaging and AFP levels after three months. We believe this strategy optimizes post-transplant recurrence risk assessment and ensures appropriate patient selection.

For patients with infiltrative HCC or those exceeding the UCSF criteria, our primary approach involves tumor biopsy and attempts to downstage primarily via transarterial interventions. These measures aid in determining prognosis by assessing “tumor biology” by response to liver directed therapy and whether new HCC tumors rapidly develop. These liver directed therapies also aim to control the primary disease. If the biopsy confirms poorly differentiated HCC, a non-transplant strategy focusing on liver directed therapies and long-term disease control appears to be the most appropriate course of action (29). Conversely, for patients with moderately or well-differentiated tumors, continued surveillance with liver directed therapy and AFP level monitoring can guide future LT candidacy decisions.

Until a decade ago, portal vein tumor thrombus (PVTT) was considered an absolute contraindication for LT. However, emerging evidence has demonstrated favorable post-transplant outcomes in select patients with PVTT, leading to its reclassification as a relative contraindication (2). Notably, radiation-based treatments, most commonly TARE, have significantly contributed to improved prognostic outcomes in this subset of patients.

Upon initial evaluation of an HCC patient in our LT clinic, the primary objective following completion of imaging and laboratory assessments is to determine whether any absolute contraindications to LT exist. Extrahepatic HCC metastases and hepatic vein tumor thrombus (HVTT) remain the most definitive contraindications to LT. In patients with HVTT, liver directed therapy is integrated into our treatment protocol as the preferred therapeutic strategy. Notably, although rare, cases in the literature describe successful outcomes following initial radiation-based bridging/downstaging, serial radiographic monitoring and eventually LT in select HVTT patients that favorably respond to liver directed therapy. These paradigms highlight the importance of close monitoring and individualized treatment planning.

In addition to these considerations, for patients meeting surgical eligibility for LT but lacking a living donor and facing a waiting period exceeding three months, we advocate for the implementation of liver directed therapies (30). The choice of intervention is tailored to individual patient characteristics based on previously outlined criteria. For lesions <3 cm, treatment selection between RFA and MWA is guided by lesion localization and IR physician device experience. For lesions >3 cm, treatment selection between TARE and TACE is determined by the patient's hepatic functional reserve. Furthermore, in cases of multiple tumors, combined transarterial and thermal ablation strategies may be considered to optimize therapeutic efficacy.

### The impacts of pretransplant locoregional therapies on postoperative outcomes

Pretransplant locoregional therapies (LRTs) serve a critical role in the management of HCC patients awaiting liver transplantation, primarily by providing insight into tumor biology and aiding in patient selection. While LRTs themselves do not uniformly improve post-transplant survival, they act as a biological “stress test” that identifies favorable tumors with indolent behavior and excludes those with aggressive features. A national cohort study by Desai et al. reported no significant differences in five-year overall or recurrence-free survival between patients who received LRT and those who did not (31).

Importantly, patients achieving complete pathological response after LRT exhibit a markedly lower risk of post-transplant recurrence, underscoring the prognostic value of treatment response (32). In contrast, patients requiring multiple (≥5) LRT sessions prior to transplantation tend to have poorer overall and recurrence-free survival, reflecting more aggressive disease and potential microvascular invasion (33). Consequently, the intensity and frequency of LRT, rather than its mere application, may provide important prognostic information.

The type of LRT also appears relevant. Although TACE has been the most widely used modality, multimodal approaches such as TACE combined with TARE have been associated with higher rates of tumor necrosis on explant pathology (34, 35). These findings suggest that more effective local tumor control prior to transplantation may translate into lower recurrence risk, although definitive survival benefits remain to be fully established.

Finally, the emerging use of immune checkpoint inhibitors in patients on the transplant waiting list introduces additional complexity. While these therapies can elicit robust radiologic and pathologic responses, they have been associated with increased risks of acute rejection if transplantation occurs too soon after treatment, highlighting the importance of appropriate timing and washout periods (36). Collectively, the evidence indicates that pretransplant LRT influences post-transplant outcomes by revealing tumor biology and guiding patient selection rather than exerting a uniform protective effect across all recipients.

## Conclusion

Liver directed therapy plays a crucial role in the management of HCC patients being considered for LT. Through downstaging, these oncologic interventions enable patients initially deemed ineligible for the waiting list to meet LT HCC tumor criteria and gain access to the waitlist. Meanwhile, bridging therapies help prevent tumor progression in already waitlisted patients, reducing the risk of dropout and significantly improving overall survival. The benefits of these approaches extend beyond primary tumor control; they also allow sufficient time for the detection of previously undiagnosed metastatic lesions during the waiting period, ensuring that LT is performed with appropriate indications and optimizing long-term outcomes.
